# Psychosocial factors related to the behavioral intention of people with type 2 diabetes using insulin

**DOI:** 10.1590/0034-7167-2021-0617

**Published:** 2022-12-16

**Authors:** Bernadete de Lourdes André Gouveia, Mailson Marques de Sousa, Taciana da Costa Farias Almeida, Wallison Pereira dos Santos, Danilo Donizetti Trevizan, Maria Júlia Guimarães Oliveira Soares, Simone Helena dos Santos Oliveira

**Affiliations:** I Universidade Federal de Campina Grande. Campina Grande, Paraíba, Brazil; II Universidade Federal da Paraíba. João Pessoa, Paraíba, Brazil; III Universidade Federal de São João Del-Rei. Divinópolis, Minas Gerais, Brazil

**Keywords:** Intention, Attitude, Insulin, Type 2 Diabetes *Mellitus*, Behavior., Intenção, Atitude, Insulina, Diabetes *Mellitus* Tipo 2, Comportamento., Intención, Actitud, Insulina, Diabetes *Mellitus* Tipo 2, Conducta.

## Abstract

**Objectives:**

to analyze the psychosocial factors correlated with the behavioral intention of people with Type 2 Diabetes *Mellitus* (T2DM) towards insulin use.

**Methods:**

a cross-sectional study; a validated instrument based on the Theory of Planned Behavior was used to identify the direct measures (attitude, subjective norm and perceived control), indirect measures (behavioral, normative and control beliefs) and behavioral intention for the use of insulin. Descriptive analysis and Spearman’s correlation were performed for data analysis.

**Results:**

a total of 211 individuals participated in the study, with a positive median of behavioral intention. Attitude, normative and behavioral beliefs were the psychosocial factors that presented a significant correlation (r=0.16, r=-0,15 and r=0.25, respectively; p<0.05) with the intention.

**Conclusions:**

there is a positive behavioral intention in the use of insulin by people with T2DM. Attitude, normative beliefs and behavioral beliefs have a low magnitude correlation with the intention of people with T2DM to use insulin.

## RESUMO

**Objetivos:**

analisar os fatores psicossociais correlacionados com a intenção comportamental das pessoas com diabetes *mellitus* tipo 2 (DM2) para uso de insulina.

**Métodos:**

estudo transversal. Instrumento validado com base na Teoria do Comportamento Planejado foi utilizado para identificar as medidas diretas (atitude, norma subjetiva e controle percebido), medidas indiretas (crenças comportamentais, normativas e de controle) e intenção comportamental para uso da insulina. Análise descritiva e correlação de Spearman foram realizadas para a análise dos dados.

**Resultados:**

um total de 211 indivíduos participaram do estudo, com uma mediana positiva de intenção comportamental. Atitude, crenças normativas e comportamentais foram os preditores que apresentaram uma correlação significante (r=0,16, r=-0,15 e r=0,25, respectivamente; p<0,05) com a intenção.

**Conclusões:**

há uma intenção comportamental positiva no uso da insulina por pessoas com DM2. Atitude, crenças Normativas e crenças comportamentais têm uma correlação de baixa magnitude com a intenção das pessoas com DM2 de usar insulina.

## RESUMEN

**Objetivos:**

analizar los factores psicosociales correlacionados con la intención conductal de personas con diabetes *mellitus* tipo 2 (DM2) para uso de insulina.

**Métodos:**

estudio transversal. Instrumento validado basado en la Teoría de Conducta Planeada fue utilizada para identificar las medidas directas (actitud, norma subjetiva y control percibido), medidas indirectas (creencias conductuales, normativas y de control) e intención conductal para uso de insulina. Análisis descriptivo y correlación de Spearman fueron realizados para el análisis de datos.

**Resultados:**

un total de 211 individuos participaron del estudio, con una mediana positiva de intención conductal. Actitud, creencias normativas y conductuales fueron los predictores que presentaron una correlación significante (r=0,16, r=-0,15 y r=0,25, respectivamente; p<0,05) con la intención.

**Conclusiones:**

hay una intención conductal positiva en el uso de insulina por personas con DM2. Actitud, creencias Normativas y creencias conductuales tienen una correlación de baja magnitud con la intención de personas con DM2 de usar insulina.

## INTRODUCTION

The treatment of Type 2 Diabetes *Mellitus* (T2DM) is complex and requires changes in behaviors acquired throughout life that are materialized in beliefs that are difficult to modify, especially with regard to self-care^(1)^. Many people with T2DM believe that it is difficult to change their lifestyle (physical activities, weight control, healthy eating, proper use of medications, monitoring rates, reducing risks); thus, glycemic control can become ineffective and have important physical and psychological impacts, such as depression^(2-3)^. For this reason, several studies have highlighted the relevance of adherence to healthy habits, especially oral or injectable drug treatment^(4-8)^.

As part of the drug therapy, the use of insulin to control T2DM has become an increasingly common health behavior due to the high incidence of cases with uncontrolled blood sugar levels, due to the abandonment or inadequate use of oral anti-diabetic drugs (ADD), or even an insufficient response to the proposed treatment, with the emergence of negative outcomes often irreversible^(9)^.

An understanding of the factors underlying the person’s decision to be involved or not in the behavior of taking insulin becomes essential to guide the development of appropriate intervention programs to promote this behavior. Thus, the use of an appropriate theoretical framework can legitimize the identification of these factors that become quite relevant^(10)^. Research studies focused on health behaviors have used the Theory of Planned behavior (TPB)^(11)^ of to identify the determining factors of the intention to perform or not behaviors in the social and health contexts^(6,8,10)^. Studies that apply the TPB in Brazil are yet incipient. Previous evidence^(12-14)^ are identified to assess the behavioral intention and use of oral anti-diabetics. Therefore, it is justified to propose further investigation to verify the factors that may be correlated with the intention to use insulin, a drug that requires individual skills for the management, storage and administration, whether of the patient or his caregiver.

According to the TPB, the Intention (Int) is the immediate construct and main antecedent of the behavior, being determined by three direct components: Attitude (At) related to the behavior, which corresponds to favorable or unfavorable assessment of the behavior, which in turn is determined by negative or positive beliefs about it; the Subjective Norm (SN), which refers to the social pressure perceived by the individual regarding opinions of their social referents for performing the behavior, and the Perceived Behavioral Control (PBC), understood as the individual perception of the extent to which performing the behavior is easy or difficult, that is, how much is under their control to perform a certain Behavior^(11)^.

Each of these direct determinants of the intention is formed by indirect variables (specific beliefs), which are: behavioral beliefs (BBs) (related to the consequences of the behavior); normative beliefs (NBs) (confronted to the important social referents for the individual regarding the target behavior); and control beliefs (CBs) (facilitators or inhibitors of the behavior related to the control power)^(11)^.

According to the TPB, the intention is related to how much people are or, are not willing to try to perform the behavior in question and how much effort they plan to commit to perform such behavior. Thus, the intention only translates into behavior if the person decides to do it^(11)^. However, to perform the behavior in question, it is not enough to have a positive intention, but also the availability of opportunities and necessary resources, such as time, money, skills, and cooperation from third parties, among others. The combination of all these factors can contribute to the performance of the behavior^(15)^.

It is understood that drug therapy is a complex process and requires motivation, knowledge, skills, social support and adequate resources for its practice to be established in the daily lives of people with T2DM^(16)^. As a useful conceptual framework to deal with the complexities of human behavior, the TPB presents itself as a consistent theoretical model to understand the aspects involved in the use of insulin as a therapy indicated for the control of people with T2DM^(17)^.

Recently, a research study based on the assumptions of the TPB identified the beliefs of people with T2DM related to the use of insulin and evidenced several behavioral (such as keeping diabetes under control and pain in the application of insulin), normative (such as associating taking insulin in the presence of children) and control (strengths and weaknesses in applying insulin, for example) beliefs^(18)^. However, no studies were found assessing the psychosocial factors that influence the intention of people with T2DM to use insulin.

## OBJECTIVES

To analyze the psychosocial factors correlated with the behavioral intention of people with type 2 diabetes *mellitus* (T2DM) to use insulin.

## METHODS

### Ethical aspects

This study was approved by the local Research Ethics Committee. The informed consent was obtained from all the participants.

### Design and place of study

A cross-sectional study conducted in an endocrinology outpatient clinic of a university hospital linked to the Unified Health System (UHS) in a capital city in the Northeast of Brazil. The study followed the recommendations of the STROBE Statement^(19)^.

### Population, sample, criteria of inclusion and exclusion

To participate in the study, people with T2DM treated at the outpatient clinic had to meet the following criteria: being an adult (over 18 years old), receiving treatment at that outpatient clinic, and using insulin exclusively for six months. The participants were excluded if they had any neurological impairment confirmed in medical records, difficulty in understanding and verbal communication, and history of hospitalizations or surgery within less than 90 days.

The sample size was calculated from a pilot test involving ten participants from such outpatient clinic. Considering that the proportion of intention to use insulin is 90%, i.e., 0.9, with a 95% confidence interval and sampling error of 4%, the minimum sample size was defined in 211 participants.

### Study protocol

Data collection was carried out from July to October 2018, through individual interviews and consultation of medical records. On the day of data collection, after obtaining the signature of the Free and Informed Consent Term in two copies, the participants answered the instrument with questions that included socio-demographic, clinical and psychosocial variables, and the filling was performed by the researcher.

### Characterization of the participants:

The socio-demographic data were obtained by the following variables: age, gender, self-declared race, marital status, schooling in years, employment status, family income; and clinical: time since diagnosed with T2DM, last glycated hemoglobin (A1c) value, lifestyle (use of tobacco and alcohol consumption), presence of comorbidities, regular practice of physical activity, identification of the current prescription with doses used and type of insulin.

### TPB variables:

The questionnaire to measure the psychosocial factors and behavioral intention of people with T2DM for insulin use was constructed according to the TPB recommendations^(11,15)^, supported by a previous study^(18,20)^. The questionnaire to measure the psychosocial variables was created and submitted to content validity by seven specialist judges (four nurses and three psychologists) with knowledge and experience in studies involving health behaviors, T2DM and validation studies. The experts were asked to evaluate each item in terms of clarity and relevance; the Content Validity Index (CVI) was calculated to measure the proportion according to the clarity and relevance of each item of the instrument^(20)^.

Subsequently, the instrument was applied to a sample of ten participants from the target population in order to assess the understanding of the instructions, the questions and the form of the answers. For this, the criteria proposed by Tourangeau were used, namely: a) Understanding the questions; b) Recalling information from memory; c) Judgment process; and d) Response process^(21)^. After this cognitive interview, the necessary adjustments were made and the final version of the questionnaire was composed.

The instrument^(20)^, with 40 items, comprises four for the direct measures (intention, attitude, subjective norm and perceived behavioral control) and 36 for the indirect measures (14 items for behavioral beliefs; eight for normative beliefs and 14 for control beliefs), as described below:

Intention: It was measured by one item: “I intend to take insulin as prescribed in the next 30 days” (very likely - very unlikely), varying from 1 to 5 points and with a CVI of 1.00^(20)^.

Attitude: Measured by one item: “For me, taking insulin as prescribed in the next 30 days is”; and four pairs of adjective answers (Very beneficial - Very harmful; Very easy - Very difficult; Very pleasant - Very unpleasant; Very useful - Very useless), varying from 1 to 5 points and with a CVI of 1.00^(20)^.

Subjective norm: Measured by one item: “Most people who are important to me think that I should take insulin as prescribed in the next 30 days”, with a scale (very likely - very unlikely) varying from 1 to 5 points and a CVI of 0.83^(20)^.

Perceived behavioral control: Measured by one item: “Taking insulin as prescribed in the next 30 days depends only on me”, accompanied by a scale (Totally agree - Totally disagree) varying from 1 to 5 points and a CVI of 1.0^(20)^.

Behavioral beliefs: Evaluated by 14 items, seven being of behavioral beliefs, for example: “Taking insulin as prescribed in the next 30 days will keep blood sugar under control”, with a scale (Strongly agree - Strongly disagree) varying from 1 to 5 points and CVI of 1.0; and seven with assessment of the consequences, for example: “Keeping blood sugar under control when taking insulin as prescribed is:”, with a scale (Very good - Very bad) varying from 1 to 5 points and a CVI of 1.0^(20)^.

Normative beliefs: Measured by eight items, of which four are normative beliefs, for example. “My children think that I should take insulin as prescribed in the next 30 days”, with a scale (very likely - very unlikely) varying from 1 to 5 points and a CVI of 0.83. And four of motivation, for example: “When it comes to taking insulin as prescribed, I do what my children think I should do”, accompanied by a scale (Totally agree - Totally disagree) varying from 1 to 5 points and a CVI of 1.0^(20)^.

Control beliefs: They were measured by 14 items, of which seven were control beliefs, for example: “I will have no trouble using insulin as prescribed in the next 30 days”, with a scale (very likely - very unlikely) varying from 1 to 5 points and a CVI of 1.0. And seven items of control power, for example: “The ease of using insulin will allow me to follow the treatment prescribed in the next 30 days”, with a scale (Totally agree - Totally disagree) varying from 1 to 5 points and a CVI of 1.0^(20)^.

### Analysis of results and statistics

The data was entered into an electronic spreadsheet and then transferred to a statistical package to perform the following analyses:

Descriptive: to describe the socio-demographic and clinical characteristics, as well as the scores of the psychosocial variables. The Anderson-Darling test was used to assess the normality of the behavioral variables, showing that they did not have a normal distribution; therefore, non-parametric tests were adopted. The scores of the TPB behavioral variables were calculated by constructs and evaluated separately. For the direct measures (intention, attitude, subjective norm and perceived behavioral control), the score was obtained by the mean of the items. For the indirect measures, the product of the beliefs was calculated as follows: the higher the scores, the greater the motivation to perform the behavior. Thus, the scores of the behavioral beliefs ranged from 7 to 175 (strength of behavioral belief *x* assessment of the consequences); of the normative beliefs, from 4 to 100 (strength of normative belief *x* the motivation to agree with the referent); and of the control beliefs, from 7 to 175 (strength of the control belief *x* control power)^(20)^.

Correlation: The Spearman’s Correlation Coefficient was applied to analyze the correlation between the variables that make up the TPB model (predictor variables) and the Intention (outcome variable). The magnitude of the correlations was based on the following criteria: <0.30 considered to be of low magnitude; between 0.30 and 0.50, of moderate magnitude; and >0.50, of strong magnitude^(22)^.

## RESULTS

A total of 211 participants undergoing exclusive insulin treatment were included in this study. Table 1 presents the socio-demographic and clinical characteristics of the participants.

**Table 1:** Socio-demographic and clinical characteristics of people with Type 2 Diabetes *Mellitus* using insulin, João Pessoa, Paraíba, Brazil

Characteristics of the participants	
Age (years old), mean (SD)	54.8 (13.4)
Female (n), (%)	148 (70.1)
Marital status (n), (%)	
Has a partner	129 (61.1)
No partner	82 (38.9)
Non-white (n), (%)	146 (69.2)
Schooling (years), median (min.-max.)	8.3 (0-20)
Occupation (n), (%)	
Works	183 (86.7)
Does not work	28 (13.3)
Monthly family income (in minimum wages) ^†^	
< 1	13 (6.2)
1 to 2	139 (65.9)
2 to 3	29 (13.7)
Associated clinical conditions (n), (%)	
Hypertension	156 (73.9)
Others (depression, hypothyroidism, osteoporosis, neuropathies and diabetic foot)	108 (51.2)
Time with DM2 (years), mean (SD)	13.4 (8.7)
HbA1c, median (min.-max.)	8.2 (5.2-14.7)
Types of insulin (n), (%)	
Fast-acting and intermediate-acting associated (Regular and NPH)	101 (47.9)
Intermediate (NPH)	97 (46.0)
Other insulins	13 (6.1)

^†^Equivalence on 14/06/2022: 1 Minimum wage (MW) = R$ 1.212.00 (USD 236,28); US$ 1.00 = R$ 5,13.

Table 2 shows the median and interquartile ranges of the behavioral variables (direct and indirect measures) of the TPB. In this table, the median of the relevant intention is observed and positively motivated to perform the behavior. The scores obtained show a positive tendency to perform the behavior, except for the normative beliefs (and the motivation to agree with social referents), which are very different from the direct measurement of the subjective norm.

**Table 2:** Presentation of the direct and indirect measures of the intention towards insulin use in people with Type 2 Diabetes *Mellitus*, João Pessoa, Paraíba, Brazil

Behavioral Variables	Median	Interquartile Range^*^
Attitude (At.)	3.5	3.12 - 4.00
Behavioral Beliefs (BBs)	111.0	96.00 - 126.50
Subjective Norm (SN)	5.0	4.00 - 5.00
Normative Beliefs (NBs)	27.0	15.00 - 41.00
Perceived Behavioral Control (PBC)	5.0	5.00 - 5.00
Control Beliefs (CBs)	120.0	97.00 - 144.50
Intention (Int.)	5.0	5.00 - 5.00

* *Interquartile range (25-75).*

Table 3 presents the correlations of the direct and indirect measures with the behavioral intention. A positive correlation is observed between intention and attitude (r = 0.16; p < 0.01) and between intention and the normative beliefs (r = 0.25; p < 0.05), although with a weak magnitude. Higher attitude and normative beliefs scores can collaborate to increase the intention towards insulin use.

**Table 3:** Correlation between the direct and indirect measures and the behavioral intention towards insulin use in people with Type 2 Diabetes *Mellitus*, João Pessoa, Paraíba, Brazil

Variables	At.	BB	SN	NB	PBC	CB
Int.	0.16^*^	-0.15^*^	0.11	0.25^*^	-0.03	0.13
At	1.00	-0.20**^+^**	0.05	-0.01	0.07	0.27^*^
BB	--	1.00	-0.06	0.02	0.08	-0.03
SN	--	--	1.00	0.27^*^	-0.13	-0.06
NB	--	--	--	1.00	-0.23^*^	-0.21^*^
PBC	--	--	--	--	1.00	0.34^*^
CB	--	--	--	--	--	1.00

* *p<0.05. Int. - Intention; At. - Attitude; BB - Behavioral Beliefs; SN - Subjective Norm; NB - Normative Beliefs; PBC - Perceived Behavioral Control; CB - Control Beliefs.*

## DISCUSSION

This study analyzed psychosocial factors related to the behavioral intention of people with DM2 for the use of insulin in a capital of northeastern Brazil. The socio-demographic and clinical characteristics of the participants were similar to that of previous studies that used TPB as a theoretical model in a study involving people with DM2^(12,18,23)^. Low schooling reflects the reality of Brazilian adults; this can be a barrier for health services to be able to offer educational guidance for self-care of glycemic control in order to generate behavioral changes^(24)^.

Despite the low level of education and income, the participants had a median situated in the highest level of agreement for the intention to use insulin to control T2DM in the next 30 days. It was identified that attitude and normative beliefs were positively related to intention. Although with low intensity, the correlation draws the attention to the need to enhance educational resources with an interest in the attitudinal components, that is, that taking insulin is beneficial, easy, pleasant and useful, as well as in the rules (strength of positive referents and motivation to perform what the referents consider important), since the increase in these variables presents a potential contribution to the increase in behavioral intention.

Regarding the analysis of the Attitude direct measure, the participants answered that, although the use of insulin is easy and beneficial, they also considered it unpleasant and even useless, as observed in the positive median, but located very close to the neutral point of the scale. This finding suggests that, while the participants recognized the benefit and ease of use of insulin, they considered the therapy unpleasant and even useless, which can be a contributory factor so that the median does not approach better scores on the scale. The discomfort can be attributed to the pain caused by the needle, hypoglycemia, and spots and hardening at the application site, which can happens several times a day, depending on the glycemic levels that the patient has during the treatment. If these values are persistently high with the use of insulin, the participants will always consider the treatment adopted useless, even if they place the control of the disease only in the behavior of using the medication. Other studies too report similar complaints of discomfort^(25-26)^.

The normative beliefs showed medians that are on the negative side of the scale, that is, when individually positioning each referent identified (son, husband, mother and brother) and associating it with the individual’s motivation to perform the behavior based on what the referents thought, many participants tended to disagree. This finding refers to the consideration that the use of insulin was not a prescribed or ordered behavior by important people directly linked to those surveyed, but something inherent to those who considered it, but that could and should have social support.

This finding differs from a previous study^(27)^ and reveals that there was a greater chance of performing the behavior among those who lived with a partner and children. Thus, it is important to highlight the role of the facilitators or inhibitors of this practice, whether physical or not, which does not depend on whether the individual follows the opinion of the family members regarding the treatment, although not ignoring the challenge faced by the patients who do not have social support when they need knowledge and skills for the correct application of insulin.

Furthermore, when considering that taking insulin is a personal decision, that it will be easy to apply and that there will be free distribution, important motivational aspects are added that can increase the chances of achieving greater adherence to the use of insulin. It is considered that barriers to treatment, such as difficulty to apply, resistance to accept the treatment and pain when applying insulin, are interference factors that can be solved with the support of social referents and reinforcement of positive beliefs about the beneficial action of insulin.

With regard to the referents, even if patients are independent in insulin self-administration, it is important that they are sensitive to the delicacy of having a loved one dependent on the use of insulin, encouraging self-care and being solicitous for any future need, so that undesirable symptoms are prevented and the benefits of glycemic control and well-being are achieved by the correct use of insulin.

There was also a negative correlation of low intensity, but not less important, of the behavioral beliefs with the intention. The contribution of the negative beliefs regarding insulin therapy is attributed to this finding, since the more people with diabetes agree that taking insulin will cause discomfort due to the pain of application, hypoglycemia, mild bleeding, purplish spots and hardening at the application site, the less they will have the behavioral intention to carry out the treatment.

For this reason, adequate guidance and training of the patients for self-administration or of those responsible for care are of substantial importance, in view of the potential to minimize discomfort due to mistakes in the preparation and administration of insulin. Therefore, the individualized attention of the health professionals to people with diabetes is considered essential, minimizing errors and difficulties of this population in self-care behaviors^(24,28)^.

A research study carried out in Japan showed that 91% of the participants reported that it is easy to prepare insulin doses. However, there were difficulties related to the obligation and lack of flexibility of insulin therapy, as they needed to inject insulin daily, even when traveling and when leaving their homes, and they felt it was embarrassing to use it in public. In that study, 76% of the medical professionals participating answered that it was difficult for the patients to prepare insulin injections^(29)^. Unlike this finding, the control beliefs related to the barriers to the intention to perform the behavior were difficulty to apply, resistance to accept the treatment, acquisition of insulin by purchase, and pain to apply. The need to eliminate or minimize such barriers to the use of insulin is inferred, understanding that the suspension of the prescribed treatment should not be an option for people with T2DM on exclusive use of insulin.

These findings refer to the complexity of insulin therapy, which people undergoing treatment do not expect only to improve their health, feel good, control blood sugar levels and not to need to take oral anti-diabetic drugs, but also believe in the importance of social support to encourage, guide and assist in insulin application. Performing a behavior favorable to one’s own health points to the need for help from the social network, with success in the continuity of treatment. Thus, health education strategies should be instituted in order to model negative beliefs into positive, verified in a previous study on the beliefs of insulin use^(18)^.

### Study limitations

This study has as a limitation the infeasibility of more robust analyses, such as regressions or generalized linear models, to verify which of the variables in the theoretical model could predict the intention, considering that the measure of intention was positive in the entire sample. The results found cannot be generalized, since the results are cross-sectional and from a single center. It is recommended that additional research studies be carried out to deepen the studied behavior and its predictive variables, since it is a relevant theme and little explored in the health context.

Finally, it is worth noting that the TPB has a specific methodology to formulate the measurement instrument, which should be built based on the beliefs that guide the behaviors of interest in the context that it is intended to investigate, and that instruments prepared based on the literature may not include all the beliefs relevant to a specific context. Therefore, instruments applied in culturally distinct regions may not prove to be reliable and adequate, since it is important to obtain the beliefs of a layer representative of the population of interest before deciding to use those prepared for other realities. Although the construction of the instrument has met the recommendations of the theoretical framework, a future research will be developed to refine it through exploratory and confirmatory factor analysis tests and its relationship between the measured indicators.

### Contributions to the fields of Nursing, Health or Public Policy

These findings are relevant as they provide information to health professionals, especially nurses, to understand the psychosocial factors associated with the use of insulin and, thus, to outline specific interventions that involve the constructs to enhance and maintain the performance of the behavior in accordance with the therapeutic recommendations.

## CONCLUSIONS

There is positive behavioral intention for the use of insulin in people with T2DM. Attitude, Normative Beliefs and Behavioral Beliefs have a low magnitude correlation with the intention of people with T2DM to use insulin.
